# Can fungal endophytes suppress *Trialeurodes vaporariorum* and the transmission of tomato infectious chlorosis and chlorosis viruses in field conditions?

**DOI:** 10.3389/fcimb.2025.1470821

**Published:** 2025-02-04

**Authors:** Marial Makur Zechariah Paweer, Everlyne Samita Namikoye, Shem Bonuke Nchore, Komivi Senyo Akutse

**Affiliations:** ^1^ International Centre of Insect Physiology and Ecology (icipe), Nairobi, Kenya; ^2^ Department of Agricultural Science and Technology, Kenyatta University, Nairobi, Kenya; ^3^ Unit of Environmental Sciences and Management, North-West University, Potchefstroom, South Africa

**Keywords:** *Trichoderma asperellum*, *Hypocrea lixii*, *Trialeurodes vaporariorum*, tomato infectious chlorosis virus, incidence, severity, tomato chlorosis virus, colonization

## Abstract

Field trials were conducted for two seasons in two experimental sites (Mwea in Kirinyaga and Ngoliba in Kiambu counties of Kenya) to assess the efficacy of fungal endophytes *Hypocrea lixii* F3ST1 and *Trichoderma asperellum* M2RT4 in the control of *Trialeurodes vaporariorum* vector of tomato infectious chlorosis virus (TICV) and tomato chlorosis virus (ToCV) through seeds inoculation. TICV and ToCV’s disease incidence, severity and the yield were also evaluated. All the fungal endophytes successfully colonized all the tomato plant parts, but the highest root colonization was observed in *H. lixii* F3ST1 compared to the *T. asperellum* M2RT4 in both seasons. The number of nymphs was significantly lower in the endophytically colonized tomato plants than the control treatments in all the seasons and at both sites. However, the lowest number of nymphs was recorded in *H. lixii* F3ST1 compared to *T. asperellum* M2RT4. On the other hand, the TICV and ToCV disease incidence and severity rates were lower in endophytically colonized tomato crops compared to the control plots. This could be attributed to the reduction in the virus replication and lower feeding ability of *T. vaporariorum* that was characterized by less excretion of honeydew causing sooty mold. However, no significant difference was observed in ToCV disease severity rates among the treatments and across the seasons. The yield was significantly higher in endophyte plots than the control treatments in both sites and across the two seasons. This study demonstrates that *H. lixii* F3ST1 and *T. asperellum* M2RT4 endophytically colonized tomato plants and conferred systemic resistance against *T. vaporariorum* vector, and significantly reduced the transmission of TICV and ToCV, contributing to high reduction of both diseases’ incidence and severity in the field. However, further studies are warranted to confirm these results at large scale trials.

## 1 Introduction

Tomato is the second-most produced and valuable/consumed vegetable after potato, accounting for up to 20.1% of the total vegetables production, and belongs to Solanaceae family (Horticultural Crops Directorate, 2019). According to epidemiological studies, there is lower risk of cancer and cardiovascular disease when tomato is consumed raw (Clinton, 1998; Giovannucci et al., 2002). On the other hand, tomato bioactive components have antioxidant properties and have been credited with protective functions (Borguini and Torres, 2009). However, tomato production is constrained by pests and diseases that cause high yield loss. In Africa, significant yield loss ranged from 11 to 43% on average, but could reach 100% in some areas due to both direct and indirect effects of insect pests and diseases on tomato (Rwomushana et al., 2019). Among the insect pests that threatened tomato production and productivity, whitefly is ranked as one of the major well-known sucking pests which causes significant crop damage and yield losses as result of nymphs and adults feeding and which also transmits viruses (Abubakar et al., 2022).

The greenhouse whitefly, *Trialeurodes vaporariorum* Westwood (Hemiptera: Aleyrodidae), is one of the major pests of horticultural crops and causes significant damage to both open-field and protected crops (Wang et al., 2017). *Trialeurodes vaporariorum* has massive spread on tomato and is the most common and dominant species in various agroecological zones of Kenya (Khamis et al., 2021). Both adults and nymphs of *T. vaporariorum* damage the plant by feeding on the phloem, which causes significant nutrients lost or deficiency, and consequently lower plant productivity (Arnó i Pujol et al., 2009; Gao et al., 2017). In crops like tomato, *Solanum lycopersicum*, the honey-dew excreted by whiteflies during feeding also promotes the growth of sooty mould, which discolors and reduces the quality of harvestable/marketable fruits *(* Anderson et al., 2005). *Trialeurodes vaporariorum* not only infest the crops directly but also indirectly cause significant economic damage by spreading a number of plant viruses such as,the tomato infectious chlorosis virus (TICV) and the tomato chlorosis virus (ToCV) (Navas-Castillo et al., 2011). The pest transmitted viruses losses hinge on the viral type, disease’s prevalence, and developmental phase of the crop (Lapidot et al., 2014). Tomato infectious chlorosis virus symptoms include interveinal chlorosis, interveinal leaves portions turn brilliant yellow as symptoms worsen but the veins are ever green (Hartono et al., 2003); whereas in tomato chlorosis virus, older leaves have light green polygonal spots that lead to interveinal yellowing, which resembles a magnesium deficiency. Within the yellowed sections, bronzing and red patches are common (Louro et al., 2000).

To manage the greenhouse whitefly, *T. vaporariorum* transmitted plant viruses, growers rely on vector management mostly through chemical pesticide applications (Whitfield et al., 2015). However, frequent use of pesticides has negative impacts on the dynamics of whitefly vector populations and lead to quick development of resistance as well as resurgence of pesticide resistant whitefly populations (Denholm et al., 2003). Furthermore, thick whitefly’s cuticular layer prevents active ingredients from penetrating the insect’s cuticle, which leads to the difficulty of controlling them in the field using pesticides (Patel et al., 2001; James et al., 2003). Moreover, this difficulty in managing the pest is also aggravated by whitefly’s cryptic behavior (Wang et al., 2017). It is established that alternative hosts diversity provides safety for insects evading pesticide applications, and some insects receiving sub-lethal doses. These insects may therefore carry resistance genes and viruses from different taxonomic groups, thus, new disease complexes developed (Gilbertson et al., 2015).

In addition, synthetic pesticides application in controlling the whitefly has negative effects on the environment, biodiversity, public/human health, and toxic effect to the pollinators as well as chemical residues in the harvested products (Abubakar et al., 2022). Therefore, exploring some agroecologically safe and sustainable approaches as alternatives could overcome the synthetic pesticides drawbacks (Kim et al., 2014). As a result, biological control approaches using microorganism-based products (microbial-based biopesticides) and parasitoids or predators become more sustainable alternatives to be used and promoted in different cropping systems (Naranjo, 2001; Jaber et al., 2018; Sani et al., 2020; Akutse et al., 2020). In addition, endophytes, key microscopic organisms that live inside their host plants without causing any disease symptoms, are also among biological agents that induce protection of their hosts against herbivorous and diseases (Vega et al., 2008; Poveda, 2021b). They have been demonstrated to cause improved protection against herbivorous insects through the induction of systemic resistance, most likely as a result of changes in biochemistry and physiology of the associated host plants (Rostás et al., 2006; Pineda et al., 2012). Biocontrol agent such as *Encarsia formosa* (Hymenoptera: Aphelinidae) is a well known host-specific parasitoid of *T. vaporariorum* but its application at a larger scale was significantly hampered by the abusive use of synthetic chemicals. In addition, it was also established that the efficacy of entomopathogenic fungus *Beauveria bassiana* in controlling insect pests like the greenhouse whitefly highly depends on the environmental factors such as temperature and relative humidity, and its pathogenicity/virulence level is pests specific. Paradza et al. (2021b) previously demonstrated that the fungal endophytes *Hypocrea lixii* F3ST1 and *Trichoderma asperellum* M2RT4 endophytically colonized *S. lycopersicum* and suppress *T. vaporariorum* nymph development and adult emergence. Therefore, this study explored the validation of these findings under field conditions. In addition, the authors also hypothesized that the suppression of *T. vaporariorum* vector could also reduce the viral diseases transmitted by the pest. The current study was therefore designed to explore the efficacy of these two fungal endophytes in controlling the *T. vaporariorum* vector and their potency in reducing tomato infectious chlorosis virus and tomato chlorosis virus incidence and severity under field conditions.

## 2 Materials and methods

### 2.1 Study sites and field preparation for planting

Field trials were conducted at Ngoliba (Kiambu county) and Mwea (Kirinyaga county), Kenya with geographical references of 1° 04’ 22.8”S 37°18”19.0”E, 1399 m a.s.l and 0° 37’7.35”S 37° 22’.973 “E, 1159 m a.s.l, respectively, for two consecutive seasons: season one (October 2022 – February 2023) and season two (April – August 2023). The field site in Mwea had deep and moderately fertile volcanic soils while Ngoliba has Pellic vertisol soil type. The experimental fields were prepared by slashing, tilling and harrowing to obtain fine tilth. Each of the experimental field was set at 21 cm × 29 m plots and was sub-divided into 28 experimental units of 4 m × 3 m each; with a path of 1 m between them.

### 2.2 Fungal cultures and insecticide

The fungal endophytes, *Trichoderma asperellum* M2RT4 and *Hypocrea lixii* F3ST1 which were previously reported to colonize tomatoes (Agbessenou et al., 2020; Paradza et al., 2021a) were obtained from International Centre of Insect Physiology and Ecology (*icipe*)’s germplasm at Arthropod Pathology Unit. The two endophytes were also reported to suppress *T. vaporariorum* nymph’s population and adult emergence. *Trichoderma asperellum* M2RT4 and *H. lixii* F3ST1 were cultured on Potato Dextrose Agar (PDA), while Sabouraud Dextrose Agar (SDA) was used to culture *Metarhizium anisopliae* ICIPE 18 which was also proved to be effective against *T. vaporariorum* adults and nymphs through inundative application (Paradza et al., 2021b). The culture plates were incubated at 25 ± 2°C in complete darkness.

In addition, Buprofezin insecticide, recommended insecticide according to the Pest Control Products Board (PCPB-Kenya) used in this study was obtained from an Agrovet (Nairobi, Kenya). Buprofezin insecticide was selected because of its effectiveness against the whiteflies and commonly used by farmers (Sain et al., 2021).

### 2.3 Mass production of fungal isolates and viability assessment

For the field trials against the *T. vaporariorum* vector and targeted diseases, conidia of *T. asperellum* M2RT4, *H. lixii* F3ST1 and *M. anisopliae* ICIPE 18 were mass produced using Pishori grain rice on a long-rice substrate in Milner bags (60 × 35 cm). Rice substrate was autoclaved for 1 hour at 121°C, allowed to cool and inoculated with a 3-day-old culture of blastospores *T. asperellum* M2RT4, *H. lixii* F3ST1, and *M. anisopliae* ICIPE 18 in separate Milner bags (Jenkins et al., 1998). The inoculated bags were then incubated for 21 days at 20 – 26 ° C and 40 – 70% relative humidity RH). The rice substrate containing the sporulated fungal spores was then allowed to dry for five days at room temperature while in the Milner bags. Conidia were harvested by sifting the substrate through a 295-μm mesh sieve and then stored in plastic bags in a refrigerator for less than three weeks at 4 - 6°C using Opisa et al. (2018) approach.

The viability of the fungal isolates was determined before being used for field trials. The harvested conidia suspended in 10 ml distilled water with 0.05% Triton X-100 in universal bottles containing glass beads (Φ = 3 mm). The suspension was vortexed for 5 min at 700 rpm to break the conidial clumps and ensure a homogeneous suspension. Conidial concentrations were quantified using a haemocytometer under a light microscope. The conidial suspensions were adjusted to 1 × 10^8^ conidia/ml through dilution prior to field applications. For viability test, a concentration of 3 × 10^6^ conidia/ml was prepared, and 0.1 ml of the suspension was evenly spread on SDA or PDA plate and three sterile microscope cover slips were placed randomly on the surface of each inoculated plate. The plates were sealed with Parafilm and incubated under complete darkness at 25 ± 2°C. Conidia germination was assessed after 18 hours by counting 100 randomly selected conidia beneath each coverslip under a light microscope at 400× magnification. Conidia were considered to have germinated when the length of the germ tube was at least twice the diameter of the conidium (Goettel and Inglis, 1997; Inglis et al., 2012). Four replicate plates were used per isolate, and viability of each fungal isolate was determined, where approximately 96% of conidial germination was observed in all the fungal isolates.

### 2.4 Seed inoculation, seedlings maintenance and transplantation

Tomato (*Solanum lycopersicum* L. cv. “Moneymaker”) seeds were obtained from Simlaw Seeds Company Ltd., Nairobi, Kenya and were surface sterilized by washing them up successively in 70% ethanol for 2 min followed by 1.5% sodium hypochlorite for three 3 min, and finally rinsed three times in sterile distilled water. The surface sterilized seeds were placed on sterile filter paper on a clean working surface in a cabinet until the residual water evaporated. Effectiveness of the surface sterilization technique was confirmed by plating out 0.1 ml of the last rinse water onto potato dextrose agar and also imprinting of surface sterilized seeds onto PDA (tissue imprint) supplemented with 100 mg/l Streptomycin and plates were incubated at 25°C for 14 days (Schultz et al., 1998; Akutse et al., 2013). Seeds were then soaked overnight for 12 hours in conidial suspensions titrated at 1 × 10^8^ conidia ml^-1^. For the controls, sterilized seeds were soaked overnight for 12 hours in sterile distilled titrated (0.05% Triton X-100) water (Akutse et al., 2013; Agbessenou et al., 2020; Paradza et al., 2021a).

Seeds were then sown in each planting trays containing the planting substrate (mixture of manure and soil 1:5) in screen house as per the defined treatments. The substrate was sterilized in an autoclave for 2 h at 121°C and allowed to cool for 72 h prior to planting. Seeds were sowed and maintained at room temperature (25 ± 2°C, 60% RH and 12:12 L:D photoperiod). The planting trays were transferred immediately after germination to the screen house (2.8 m length × 1.8 m width × 2.2 m height) at 25 ± 2°C, 55% RH and 12:12 L:D photoperiod. Seedlings were watered twice per day (morning and evening) until when they developed 4-6 leaves. No additional fertilizer was added to the planting substrate prior to the seedlings’ transplantation.

Three weeks after germination, seedlings were transplanted at both prepared sites (Ngoliba and Mwea) on planting bed in holes which were amended with 20 g each of well decomposed goat manure mixed well with soil. Transplanting was done at a spacing of 0.6 × 0.75 m in 4 × 3 m plot giving the total number of 16 plants per plot.

### 2.5 Endophytic colonization of *Solanum lycopersicum* assessment

Endophytic colonization was determined when seedlings were three-week-old after transplanting. Seedlings (both inoculated and uninoculated) were randomly selected from each treatment, uprooted and washed under running tap water to remove any soil attached to the plants after which they were separated into different parts each (root, stem, and leaf) and cut into 1 × 1 cm pieces, and surface sterilized under a hood as described above (Jaber et al., 2018). Five fragments of each section of the plant parts were plated 4 cm from one another on PDA plates amended with antibiotics (0.25 g/l w/v chloramphenicol) (Akello et al., 2007a; Batta, 2013; Gathage et al., 2016). The plates were incubated for 10 days at 25 ± 2 °C, after which the presence of endophytes was determined. The last rinse water was also plated to assess the effectiveness of the surface sterilization procedure as described earlier. Plate imprinting was also conducted to assess effective surface sterilization of the plant materials (Schultz et al., 1998; Akutse et al., 2013). The colonization of the different plant parts was recorded by counting the number of pieces of the different plant parts that showed the presence of inoculated fungal growth/mycelia according to Koch’s postulates (Petrini and Fisher, 1986). Only the presence of endophytes that were inoculated was scored. The colonized and reisolated fungal isolates were identified morphologically using slides which were prepared from the mother plates. Treatments were arranged in a randomized complete block design (RCBD) with four replicates per experiment (Akutse et al., 2013; Agbessenou et al., 2020). The proportion of the plant parts colonized by the inoculated fungal isolate was calculated for each treatment as the number of plant pieces showing fungal outgrowth divided by the total number of plant pieces plated (Paradza et al., 2021a).

### 2.6 Experimental design, treatments and trial management

Field experimental trial was laid out in a Randomized Complete Block Design (RCBD) with seven treatments, replicated four times per season at each site. The fungal inoculated and uninoculated seedlings were transplanted as per the defined treatments. The following treatments were applied: *H*. *lixii* F3ST1-inoculated tomato seedlings (T1), *T*. *asperellum* M2RT4-inoculated tomato seedlings (T2), F3ST1- inoculated tomato seedlings + *M. anisopliae* ICIPE 18 (T3), *T. asperellum* M2RT4-inoculated tomato seedlings + *M*. anisopliae ICIPE 18 (T4), uninoculated tomato seedlings + *M*. *anisopliae* ICIPE 18 (T5), uninoculated seedlings with Buprofezin insecticide (T6), and uninoculated seedlings alone (Control) (T7). 0.75 L of Buprofezin insecticide was formulated in 15 L of water and sprayed using Knapsack twice at third and fifth weeks after transplanting. In addition to the endophytes, the *M*. anisopliae ICIPE 18 which was initially identified very effective against *T. vaporariorum* adults and nymphs, was applied in inundative application, where the fungus was formulated in olive oil (obtained from Bidco Africa Limited, Thika, Kenya) (Paradza et al., 2021b). The oil formulation was done using dry conidia that were formulated with the oil by emulsifying 2% (v/v) in 0.05% Triton X-100 water containing 1 × 10^8^ conidia/ml fungal suspension (Kirubakaran et al., 2014; Paradza et al., 2021b). In addition, the conidia were suspended in olive oil with 0.05% Integra (sticker, Greenlife Crop Protection Africa Ltd, Nairobi, Kenya) with 0.1% nutrient agar, 0.1% glycerine and 0.5% molasses added as protectants and attractants, respectively. The application was done using Knapsack sprayer at the rate of 0.15 l suspension (where 10 ml of oil-formulated product in 20 l of water) per experimental unit. Control treatment was sprayed with water containing 0.05% Triton X-100, 0.05% Integra, 0.1% nutrient agar, 0.05% molasses and 0.1% glycerine without any fungal conidia and any insecticide solution (Kabaale et al., 2022; Mweke et al., 2019). Two applications were performed at three and five weeks, respectively after transplanting. The applications were done early in the morning for two times per season.

The field experimental trial was repeated for two consecutive seasons at the two sites (Mwea and Ngoliba plots). After transplantation, watering was done using hose pipe as required and weeding was done whenever necessary using hand hoe. At the vegetative stage, booster foliar fertilizer was applied at the rate of 0.5 L in 50 L of water to help in vegetative growth of the tomato crop following the tomato farmers’ practices as per the experimental sites. Staking was done using supporting sticks and there were no fungicides or other pesticides applications throughout the experimental trials.

### 2.7 Sampling and molecular identification of the whitefly collected from the experimental sites

Whiteflies were randomly sampled from the experimental field sites. During the sampling whiteflies were collected from the underside of the leaves using camel brush. Twenty (20) whiteflies were randomly selected and placed in universal bottles containing 95% ethanol and transported to the laboratory where they were stored at -20 °C. Prior to DNA extraction, each of the insect was surface sterilized in 3% Sodium hypochlorite and rinsed three times with sterile distilled water. The genomic DNA was extracted using isolate II genomic DNA Kit (Bioline, London, UK), following the manufacturer’s instructions (Khamis et al., 2021). The purity and concentration of the resultant extracted DNA was determined using Nanodrop 2000/2000c Spectrophotometer (Thermo Fischer Scientific, Wilmington, USA) then stored at − 20°C for used in downstream processes. Polymerase chain reaction (PCR) was done to amplify a portion of the 16S ribosomal RNA (rRNA) gene region using WF-F (50 -CGCCTGTTTAACAAAAACAT-30) and WF-R (50 - CCGGTCTGAACTCAGATCACGT-30) primers (Alhudaib et al, 2014; Frohlich et al., 1999). The PCR was carried out in a total reaction volume of 20 μL containing 5X My *Taq* Reaction Buffer (5 mM dNTPs, 15 mM MgCl2, stabilizers and enhancers) (Bioline, London, UK), 0.5 pmol μl−1 of each primer, 0.5 mM MgCl2, 0.0625 U μl−1 My *Taq* DNA polymerase and 15 ng μl−1 of DNA template. This reaction was set up in the Mastercycler gradient Nexus thermal cycler (Eppendorf, Hamburg, Germany). The following cycling conditions were used: initial denaturation for 2 min at 95°C, followed by 40 cycles of 30 s at 95°C, 40 s annealing and 1 min at 72°C, then a final elongation step of 10 min at 72°C. The target gene region was 650–700 base pairs. The amplified PCR products were resolved through a 1.2% agarose gel. The DNA bands on the gel were analyzed and documented using KETA GL imaging system trans-illuminator (Wealtec Corp, Meadowvale Way Sparks, Nevada, USA). Successively amplified products were excised and purified using Isolate II PCR and Gel Kit (Bioline) following the manufacturer’s instructions. The purified samples were shipped to Macrogen Inc. Europe Laboratory, Amsterdam, the Netherlands, for bi-directional sequencing. The target species from both study sites were identified to be *T. vaporariorum* and the sequences have been submitted/deposited to GenBank, a database maintained by the National Center for Biotechnology Information (NCBI) under accession numbers OK500119.1, MW60307.1, NC_006280.1.

### 2.8 *Trialeurodes vaporariorum* population densities

Weekly samplings were conducted to determine *T. vaporariorum* population densities. Five plants were randomly selected and tagged using masking tape in each plot/unit. Using a magnifying lens, *T. vaporariorum* nymphs were counted from two lower, two middle, and two upper leaves of each plant by turning over the leaves because nymphs cluster on the undersides. Leaves were marked with mark pen to avoid sampling the same leaves many times, and a different marker was used in each sampling point. The total number of nymphs was recorded and averaged for five weeks during the vegetative stage of the crop.

### 2.9 Assessment of tomato infectious chlorosis and tomato chlorosis disease incidence and severity

The disease incidence and severity symptoms of both viruses were determined visually in all the experimental plots/units. The percentage of each disease incidence was determined by counting the total number of diseased plants in each plot divided by the total number of plants in the plot/unit, multiplied by hundred. On the other hand, disease severity was determined using a ranking scale of 0 – 5 as described by Nelson et al. (1999)with 0- there are no signs or symptoms of the disease; 1- mild mottling; 2- mottling on the leaf area/light downward cupping; 3- pronounced downward or upward leaf chlorosis leaf mottling; 4- severe mosaic/leaf distortion/crinkled leaf/plant stunting/leaf bunching; 5- severe leaf distortion/necrosis/narrowed or shoes-string leaf.

The incidence and severity were recorded at weekly intervals and averaged during the vegetative stage of the crop time over time.

### 2.10 Identification of the tomato infectious chlorosis and tomato chlorosis viruses

To confirm the viruses causing each targeted and assessed disease, symptomatic leaves of tomato infectious chlorosis and tomato chlorosis viruses were collected and put in paper bags and brought to the library in cool boxes and were stored in – 80°C incubators. They were later ground in cold extraction buffer (0.5 M trisodium citrate 0.1% thioglycolic acid). RNA was extracted from 200 µL homogenate in line with manufacturer’s instructions. Homogenate was heated at 90°C for 5 min and RT- PCR, TOCV specific primer was designed based on end region sequence of TOCV RNA, and synthesis was done by DNA synthesis. RT-PCR was then performed using specific primers created in the coat protein (CP) gene or the HSP70 homologue gene (Jacquemond et al., 2009). Each sample was lysed using a BioSpec Mini-Beadbeater-16 high-energy cell disruptor (USA) in a sterile 1.5 ml microcentrifuge tube containing plastic beads and 500 l of lysis buffer. In the final step before elution, the column was spun dried to get rid of any unwanted leftover ethanol in the wash buffer. Thermo Fisher Scientific’s nanodrop 1000 spectrophotometer was used to measure the final elution. Nested RT-PCR assays were run under the following conditions: a first denaturizing step at 95°C for 2 min, 40 cycles divided into 30 s at 95°C,1 min at 72°C, annealing at 40 s and finally one final extension step at 72°C for 10 min. The target DNA was detected by 1.5% agarose gel electrophoresis in 0.1X TBE buffer and ethidium bromide staining of the final RT-PCR products were visualized under UV light. The cleaned samples were sent to the Amsterdam, Netherlands-based Microgen Inc. Europe Laboratory for sequencing, and the sequences were subsequently uploaded into GenBank.

### 2.11 Solanum lycopersicum yield

Tomato fruits were harvested after they have reached maturation stage, at weekly interval and the fruits were sorted into damaged, cracked, diseased and marketable categories. The average weight of both damaged, cracked, diseased and marketable fruits was taken and recorded as per each experimental unit. In addition, three yield parameters such as number of fruits, fruit weight, and yield were collected in the manner outlined by Liu et al. (2019), where the total number of fruits per experimental unit and the individual fruit weight were obtained by counting and weighing each marketable fruit. The yield (kg per experimental unit) was obtained by totaling the weight of all fruits harvested from each plant over time. The yield estimation in tons per ha was calculated using the below formula described by Ali et al. (2016):


$$\text{Yield }\left({\text{T ha}}^{-1}\right)=\;\frac{\text{Yield per experimental unit }\left(\text{kg}\right)\text{ x }10,000}{\text{Experimental unit area }\left({\text{m}}^{2}\right)\text{ x }1,000}$$

### 2.12 Data analysis

Data on fungal endophytic colonization, whitefly nymph population densities, disease severity were analyzed using Generalized Linear Model (GLM) with binomial distribution and logit link function to determine treatments and study sites effects. Where there was over-dispersion in the data a quassi- Poisson distribution was used. Means were separated using Tukey’s Honesty significance difference (Tukey’s HSD) at a significance level of P < 0.05. Data for each of the site were analyzed separately per season, however comparisons were done among the treatments. The approximate χ2 distribution of the deviance, which reflects the treatment effects in GLM, is presented as test statistics. The data on disease incidence and yield were analyzed using analysis of variance (ANOVA) and the means were separated using HSD and Student–Newman–Keuls (SNK) test, respectively. All the data were analyzed using R version 4.2.2 statistical software (R Core Team, 2022).

## 3 Results

### 3.1 Endophytic colonization of *Solanum lycopersicum*

**Figure 1:**
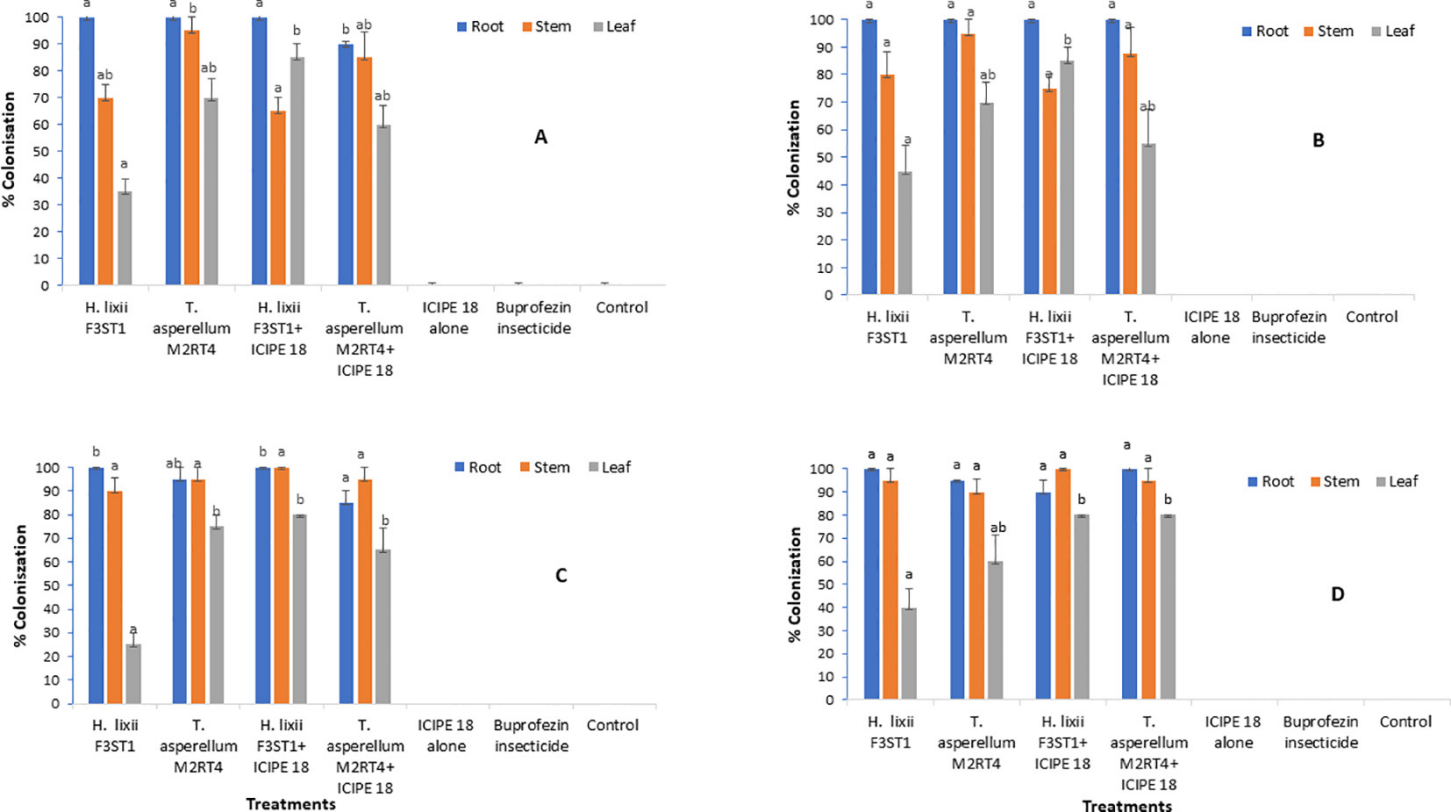
Endophytic colonization rates of the different parts of *Solanum lycopersicum* plants at Mwea **(A, C)** and Ngoliba **(B, D)** during season 1 **(A, B)** and season 2 **(C, D)**. Means followed by the same letters are not significantly different from each other at P < 0.05.

The two endophytes *T. asperellum* M2RT4 and *H. lixii* F3ST1 successfully colonized the roots, stems and leaves of the tomato plants in both sites and seasons. The colonization of the two endophytes was confirmed not only by colonization rates, but also by the presence of mycelia growth of the same inoculated fugus in comparison with the mother plates. However, no colonization was observed in the uninoculated plant treatments at the two experimental sites (**Figure 1**). In season 1, the colonization ranged between 90 - 100%, 65 - 95% and 35 - 85% for the root, stem and leaf respectively in Mwea (**Figure 1A**); while in Ngoliba 100%, 75 - 95% and 45 - 85% colonization were recorded for root, stem and leaf respectively (**Figure 1B**). At Mwea in season 1, there were significant differences in the colonization rates of the roots (χ^2^ = 8.46, df= 6, P = 0.04), stems (χ^2^ = 9.11, df = 6, P = 0.02) and leaves (χ^2^ = 13.02, df = 6, P = 0.005) among the treatments (**Figure 1A**). High colonization rates of the roots were recorded for *T. asperellum* M2RT4 (90 – 100%) and *H. lixii* F3ST1 (100%). However, *T. asperellum* M2RT4 colonized 85 – 95% of the stems compared to 65 – 70% for *H. lixii* F3ST1; while 60 – 70% and 35 – 85% colonization rates of the leaves were recorded for *T. asperellum* M2RT4 and *H. lixii* F3ST1, respectively (**Figure 1A**). Similar trends were also observed at Ngoliba in season 1 where no significant differences were observed in roots colonization among both endophyte treatments (P = 1), where the root colonization rate was 100% for both *H. lixii* F3ST1 and *T. asperellum* M2RT4 (**Figure 1B**). Similarly, no significant difference (P = 0.21) was observed in the colonization of the stems by *H. lixii* F3ST1 (75 – 80%) and *T. asperellum* M2RT4 (87.5 – 95%). However, there was significant difference (χ^2^ = 9.73, df = 6, P = 0.02) in the colonization of the leaves by *H. lixii* F3ST1 (45 – 85%) and *T. asperellum* M2RT4 (55 – 70%). No colonization was recorded for uninoculated treatment plants (**Figure 1B**).

In season 2 at Mwea, significant difference (χ^2^ = 11.54, df = 6, P = 0.009) was observed in root colonization rates where *H. lixii* F3ST1 treatments recorded 100% compared to 85 – 95% in *T. asperellum* M2RT4 treatments (**Figure 1C**). Similarly, there was significant difference (χ^2^ = 52.53, df = 6, P < 0.0001) in the colonization rates of the leaf by *H. lixii* F3ST1 treatments (25 – 80%) as compared to 65 – 75% for *T. asperellum* M2RT4 treatments (**Figure 1C**). However, there was no significant difference (χ^2^ = 2.34, df = 6, P = 0.50) in the colonization rates of the stem where *H. lixii* F3ST1 treatments recorded 90 – 100% compared to 95% for *T. asperellum* M2RT4 treatments (**Figure 1C**). Similar trends were also observed in season 2 at Ngoliba with no significant difference (P = 0.21) in root colonization rates by *H. lixii* F3ST1 treatments (90 – 100%) compared to 95 – 100% for *T. asperellum* M2RT4 treatments (**Figure 1D**). Similarly, no significant difference (P = 0.50) was observed in the colonization of the stem with *H. lixii* F3ST1 treatments recording 95 – 100% compared to *T. asperellum* M2RT4 treatment which recorded 90 – 95%. However, significant difference (χ^2^ = 18.51, df = 6, P = 0.0003) was observed in the colonization rates of the leaves where *H. lixii* F3ST1 treatments colonized 40 – 80% compared to 60 – 80% for *T. asperellum* M2RT4 treatments (**Figure 1D**). The colonization rates of the leaves observed which were lower compared to the other parts of the plant in both seasons and sites could be attributed to endophytic fungus tissue-specificity and the physiological conditions of the plants.

### 3.2 Effect of fungal endophytes on *Trialeurodes vaporariorum* nymphs*’* population densities

**Figure 2:**
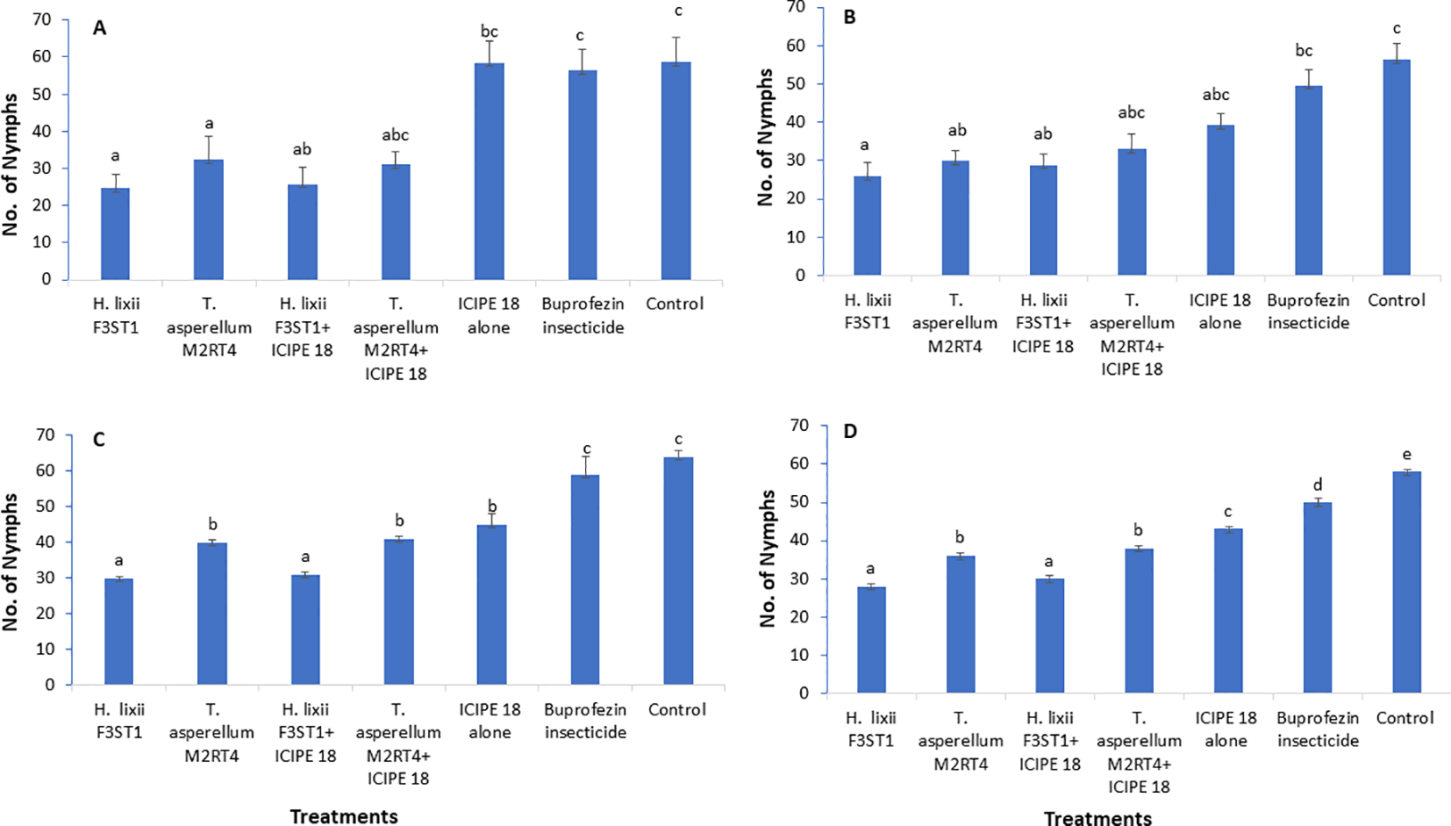
Effect of the different treatments on mean number of *Trialeurodes vaporarium* nymphs per plant within a plot/experimental unit at Mwea **(A, C)** and Ngoliba **(B, D)** during season 1 **(A, B)** and season 2 **(C, D)**. Bars represent means ± SE. Means followed by the same letters are not significantly different from each other at P < 0.05.

The two fungal endophytes *H. lixii* F3ST1 and *T. asperellum* M2RT4 suppressed the *T. vaporariorum* nymph’s population. However, there was no significant interaction between sites and treatments in season 1 (χ^2^ = 1.72, df = 6, P = 0.94). In addition, no significant difference was observed between both (Mwea and Ngoliba) sites (χ^2^ = 0.05, df = 6, P = 0.82). However, there were significant differences between the treatments at Mwea (χ^2^ = 39.62, df = 6, P < 0.0001) and Ngoliba (χ^2^ = 27.87, df = 6, P < 0.0001) regarding the *T. vaporariorum* nymphs’ population densities in season 1 (**Figures 2A, B**). The overall mean number of *T. vaporariorum* nymphs ranged between 24.70 and 58.75 and between 25.80 and 56.35 per plant in each treatment plot at Mwea (**Figure 2A**) and Ngoliba (**Figure 2B**) respectively. In season 1, fewer mean numbers of nymphs were recorded in plants inoculated with *H. lixii* F3ST1 (24.70 ± 3.8) and *T. asperellum* M2RT4 (30.75 ± 6.5) compared to the control plots (58.75 ± 6.6) at Mwea (**Figure 2A**) versus *H. lixii* F3ST1 (25.80 ± 3.6) and *T. asperellum* M2RT4 (28.85 ± 2.6) compared to control (56.35 ± 4.2) at Ngoliba (**Figure 2B**). In addition, the endophytes treated plots recorded the fewer number of nymphs in season 1 compared to buprofezin insecticide treated plots (**Figures 2A, B**).

In season 2, there was a significant difference between both (Mwea and Ngoliba) sites (χ^2^ = 18.12, df = 6, P < 0.0002). However, no significant interaction was observed between the sites and treatments (χ^2^ = 7.8, df = 6, P = 0.25). There were highly significant variations between the treatments with more *T. vaporariorum* nymphs recorded in the controls than in the endophyte treatments at Mwea (χ^2^ = 208.6, df = 6, P < 0. 0001; **Figure 2C**) and Ngoliba (χ^2^ = 1124.3, df = 6, P < 0.0001; **Figure 2D**). Moreover, the endophytes treated plots recorded the fewer number of nymphs in season 2 compared to buprofezin insecticide treated plots (**Figures 2C, D**). The number of nymphs varied between 30 and 41 and between 45 and 64 in endophyte-inoculated treatments compared to uninoculated plots, respectively at Mwea (**Figure 2C**); while at Ngoliba, the number of nymphs varied between 28 and 38 and between 43 and 58 respectively (**Figure 2D**). Fewer numbers of nymphs were recorded in *H. lixii* F3ST1 (30 ± 0.51) and *T. asperellum* M2RT4 (40 ± 0.6) compared to the control (64 ± 1.7) plots at Mwea (**Figure 2C**), versus in *H. lixii* F3ST1 (28 ± 0.7) and *T. asperellum* M2RT4 (36 ± 0.8) compared to the control (58 ± 0.5) plots at Ngoliba (**Figure 2D**).

### 3.3 Effect of fungal endophytes on incidence of tomato infectious chlorosis disease

There was no significant interaction between sites and treatments in season 1 (F_6, 42_ = 0.2, P = 0.97) and no significant variation was also observed between the experimental sites (F_1, 6_ = 3.18; P = 0.08). Significant differences were observed among the treatments at Mwea (F_6, 21 =_ 5.09, P = 0.002) and Ngoliba (F_6, 21_ = 7.24, P = 0.0002) in disease incidence. The disease incidence rates varied between 20.73 ± 0.51 and 38.16 ± 1.82% and between 18.09 ± 1.77 and 29.30 ± 2.45% in endophytically colonized treatments at Mwea and Ngoliba respectively (**Table 1**). However, the lowest disease incidence rates were recorded in *H. lixii* F3ST1 endophytically colonized plants at Mwea (20.73 ± 1.51%) and Ngoliba (18.09 ± 1.77%); while the incidence rates of 38.16 ± 3.65 and 25.2 ± 1.55% were obtained in *T. asperellum* M2RT4 endophytically colonized treatments at Mwea and Ngoliba, respectively. The highest disease incidence rates were recorded in the controls at Mwea (55.55 ± 5.83%) and Ngoliba (52.34 ± 5.18%) in season 1 (**Table 1**). Furthermore, the endophytes treated plots significantly reduced the incidence of tomato infectious chlorosis virus compared to the treatments where buprofezin insecticide was applied (**Table 1**).

**Table 1:** Effect of the various treatments on the mean percentage of tomato infectious chlorosis disease incidence during the two seasons at Mwea and Ngoliba.

Treatments	Disease incidence of tomato infectious chlorosis disease (Mean ± SE %)			
	Season 1		Season 2	
	Mwea	Ngoliba	Mwea	Ngoliba
*Hypocrea lixii* F3ST1	20.73 ± 0.51b	18.09 ± 0.77c	22.23 ± 0.23d	19.59 ± 0.03b
*Trichoderma asperellum* M2RT4	38.16 ± 1.82ab	29.30± 0.45abc	33.15 ± 0.11cd	21.55 ± 0.37b
*Hypocrea lixii* F3ST1 + ICIPE 18	22.37 ± 0.41b	20.47 ± 0.16c	24.86 ± 0.58cd	25.45 ± 0.77b
*Trichoderma asperellum* M2RT4 + ICIPE 18	36.94 ± 0.82ab	25.2 ± 0.55bc	34.44 ± 0.68c	23.22 ± 0.09b
*Metarhizium anisopliae* ICIPE 18 alone	52.98 ± 0.16a	45.16 ± 0.82ab	52.40 ± 0.31b	42.66 ± 0.99a
Buprofezin insecticide	53.27 ± 0.46a	49.82 ± 0.31a	55.77 ± 0.23ab	52.32 ± 0.55a
Control	55.55 ± 1.83a	52.34 ± 0.18a	65.55 ± 0.12a	57.34 ± 0.65a
df	6,21	6,21	6,21	6,21
F value	5.09	7.24	42.78	23.3
P value	0.002	0.0002	< 0.0001	< 0.0001

Means (± SE) followed by the same letters within a column are not significantly different at P < 0.05.

In season 2, similar trends were also observed in both experimental sites with significance differences in disease incidence among treatments at Mwea (F_6, 21 =_ 42. 78, P = 0.0001) and Ngoliba (F_6, 21 =_ 23.3, P = 0.0001). Regarding the site effects, significant difference was observed between the two sites (F_1, 6_ = 17.72; P = 0.0001), while no significant interaction was observed between the sites and treatments (F_6, 42_ = 1.26, P = 0.29). The lowest disease incidence rates were recorded in *H. lixii* F3ST1 endophytically colonized treatments at Mwea (22.23 ± 1.23%) and Ngoliba (19.59 ± 1.03%); while the incidence rates of 33.15 ± 1.11 and 21.55 ± 0.37% were obtained in *T. asperellum* M2RT4 endophytically colonized tomato crops compared to the control treatments at Mwea (65.55 ± 1.12%) and Ngoliba (57.34 ± 3.65%) respectively (**Table 1**). On the other hand, the endophytes treated plots reduced the incidence of tomato infectious chlorosis virus compared to the treatments where buprofezin insecticide was applied (**Table 1**). In general, the fungal endophytes significantly reduced the incidence of tomato infectious chlorosis viral disease and could be developed as fungal-based biopesticide to manage the incidence of the viral disease in tomato.

### 3.4 Effect of fungal endophytes on severity of tomato infectious chlorosis disease

**Figure 3:**
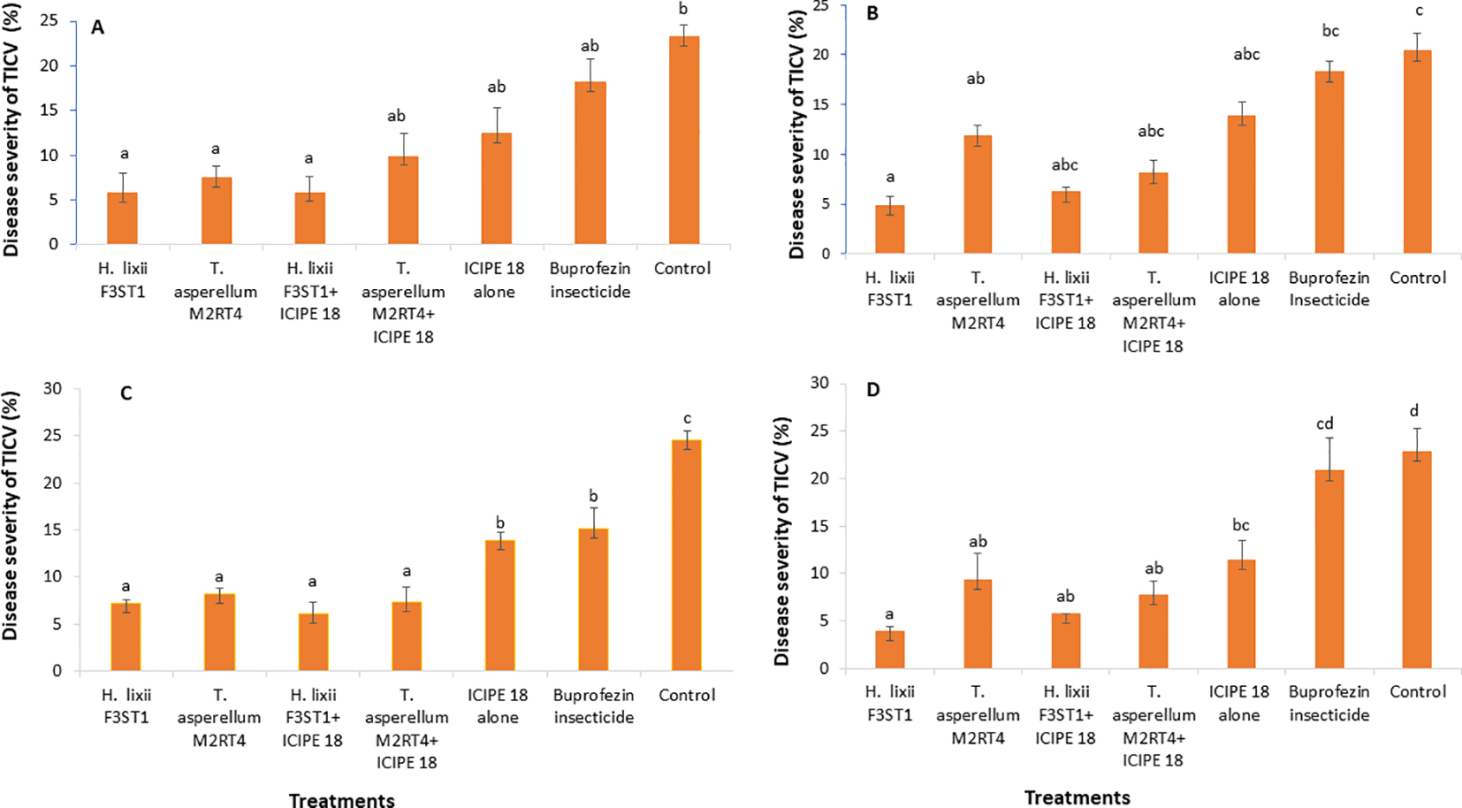
Effect of the various treatments on the mean percentage of tomato infectious chlorosis disease severity at both Mwea **(A, C)** and Ngoliba **(B, D)** during season 1 **(A, B)** and season 2 **(C, D)**. Bars denote means ± SE and means followed by the same letters are not significantly different at P < 0.05.

The fungal endophytes reduced the severity of tomato infectious chlorosis disease. In season 1, significant differences were observed in disease severity among the various treatments at Mwea (χ^2^ = 85.43, df = 6, P <0.0001; **Figure 3A**) and Ngoliba (χ^2^ = 30.49, df = 6, P < 0.0001; **Figure 3B**). However, there was no significant interaction between the sites and treatments (P = 0.89), and no significant variation was also observed between the two experimental sites (P = 0.930). The endophytically colonized *S. lycopersicon* crops had lower disease severity than the control treatments at both Mwea and Ngoliba (**Figures 3A, B**). In season 2, there was no significant interaction between both sites and the treatments (χ^2^ = 8.6, df = 6, P = 0.19). However, there were significant differences among the treatments at Mwea (χ^2^ = 129.4, df = 6, P < 0.0001; **Figure 3C**) and Ngoliba (χ^2^ = 77.1, df =6, P < 0.0001; **Figure 3D**) regarding the disease severity. For instance, the highest disease severity was recorded in the control treatments at Mwea (24.51 ± 1.04%) and Ngoliba (22.87 ± 2.39%) as compared to the endophytically colonized treatments which recorded the lowest disease severity at both sites (**Figures 3C, D**). The endophytes treated tomato crops recorded the lowest disease severity compared to the insecticide treatments which recorded the highest disease severity (**Figures 3A–D**).

### 3.5 Effect of fungal endophytes on incidence of tomato chlorosis disease

There was high reduction in the incidence of tomato chlorosis disease in the fungal endophyte plots which could be attributed to a tendency toward delay in symptoms development in inoculated plants compared to the controls. In addition, there was significant difference between the experimental sites (F_1, 6_ = 0.54; P < 0.0008) in season 1, but no significant interaction was observed between the sites and the treatments (F_6, 42_ = 1.07; P= 0.39). However, there were significant differences in tomato chlorosis disease incidence among the treatments at Mwea (F_6, 21_ = 3.41; P= 0.02) and Ngoliba (F_6, 21_ = 4.73; P= 0.003). The lowest disease incidence rates were recorded in fungal inoculated treatments compared to controls at both Mwea and Ngoliba (**Table 2**). Similarly, in season 2, there was significant difference between both sites (F_6, 42_ = 24.38; P < 0.0001) and significant interactions were also observed between the sites and treatments (F_6, 21_ = 24.38; P = 0.04). The disease incidence rates varied among the treatments with the lowest incidence recorded in *H. lixii* F3ST1 and *T. asperellum* M2RT4 endophytically colonized plots. For instance, in season 2, significant differences were observed between endophyte inoculated and uninoculated treatments at Mwea (F_6, 21 =_ 21.68; P < 0.0001) and Ngoliba (F_6, 21_ = 19.06; P < 0.0001). The disease incidence rates also varied among the treatments with the highest rates recorded in the controls at Mwea (65.58 ± 0.52%) and Ngoliba (52.21 ± 0.32%) compared to the endophyte treated plots; where the lowest disease incidence rates were recorded in *H. lixii* F3ST1 (18.88 ± 0.90 and 16.49 ± 025%) and *T. asperellum* M2RT4 (34.04 ± 0.14 and 27.57 ± 0.94%) endophytically colonized tomato crops at Mwea and Ngoliba, respectively (**Table 2**). The endophytes treated tomato crops recorded the lowest tomato chlorosis virus disease incidence compared to the insecticide treated plots which recorded the highest disease severity (**Table 2**).

**Table 2:** Effect of the various treatments on the mean percentage of tomato chlorosis disease incidence during the two seasons at Mwea and Ngoliba.

Treatments	Disease incidence of tomato chlorosis disease (Mean ± SE %)			
	Season 1		Season 2	
	Mwea	Ngoliba	Mwea	Ngoliba
*Hypocrea lixii* F3ST1	20.13 ± 0.07b	21.84 ± 0.27ab	18.88 ± 0.90b	24.34 ± 0.01c
*Trichoderma asperellum* M2RT4	36.54 ± 0.61ab	22.57 ± 0.93ab	34.04 ± 0.14b	27.57± 0.94bc
*Hypocrea lixii* F3ST1+ ICIPE 18	28.85 ± 0.00ab	18.99 ± 0.55b	26.35 ± 0.19b	16.49 ± 0.25c
*Trichoderma asperellum* M2RT4+ ICIPE 18	31.04 ± 0.21a	23.17 ± 0.31ab	36.04 ± 0.31b	28.42 ± 0.19bc
*Metarhizium anisopliae* ICIPE 18 alone	57.90 ± 0.43ab	34.80 ± 0.97ab	56.31 ± 0.26a	39.80 ± 0.35ab
Buprofezin insecticide	47.31 ± 0.16ab	36.55 ± 0.94a	57.31 ± 0.87a	44.05 ± 0.48a
Control	63.08 ± 0.87a	37.20 ± 0.12a	65.58 ± 0.52a	52.21 ± 0.32a
df	6,21	6,21	6,21	6,21
F value	3.41	4.73	21.68	19.06
P value	0.02	0.003	0.0001	0.0001

Means (± SE) followed by the same letters within a column are not significantly different at P < 0.05.

### 3.6 Effect of fungal endophytes on severity of tomato chlorosis disease

**Figure 4:**
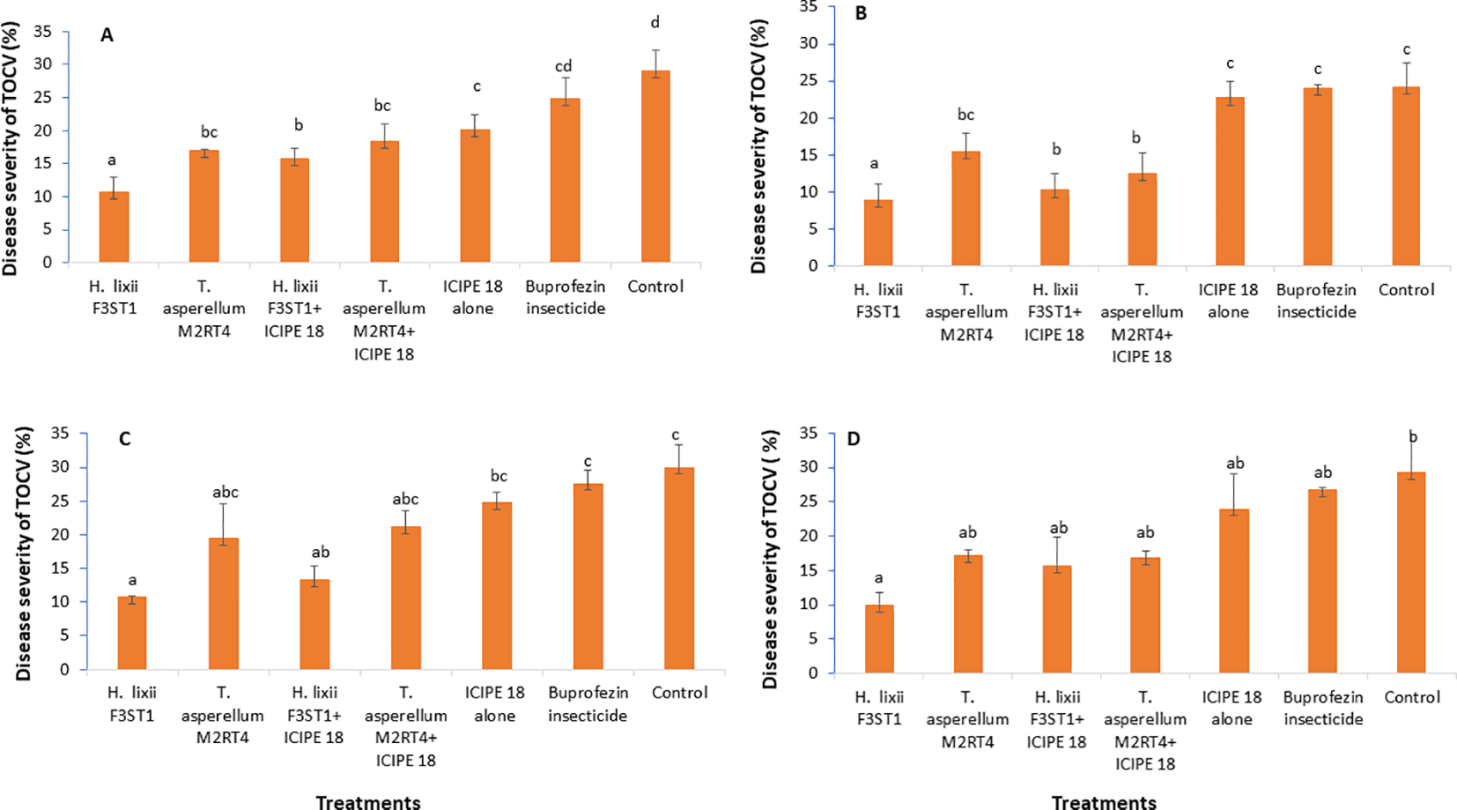
Effect of the various treatments on the mean percentage of tomato chlorosis disease severity at both Mwea **(A, C)** and Ngoliba **(B, D)** during season 1 **(A, B)** and season 2 **(C, D)**. Bars denote means ± SE, and means followed by the same letters are not significantly different at P < 0.05.

It was observed that the fungal endophytes reduced the severity of tomato chlorosis viral disease. This observed result was associated with reduced expression of viral symptoms such as leaf area reduction, leaf chlorosis, leaf mottling, severe mosaic, leaf distortion, crinkling, plant stunting and severe necrosis. There was no significant interaction between the sites and the treatments (P = 0.83) and no significant site/location effects between the two experimental sites (P = 0.41). The highest disease severity was recorded in the controls than in the endophyte’s treatments. There were significant differences between the treatments in both Mwea (χ^2^ = 37.24, df = 6, P < 0.0001) and Ngoliba (χ^2^ = 20.9, df = 6, P < 0.000) in season 1, where the percentage disease severity rates varied between 8.9 and 10.7% in endophyte treatments compared to 24.2 and 29% in the control treatments in both Ngoliba and Mwea, respectively (**Figures 4A, B**). In season 2, there was significant difference between the sites and treatments (χ^2^ = 41.62, df = 6, P < 0.0001), while no significant variation was observed between the experimental sites (χ^2^ = 0.70, df = 1, P = 0.40). In addition, there were significant differences among the treatments at Mwea (χ^2^ = 41.83, df = 6, P < 0.0001) and Ngoliba (χ^2^ = 17.28, df = 6, P = 0.008) as regard to the disease severity in season 2 (**Figures 4C, D**). The lowest disease severity was recorded in *H. lixii* F3ST1 treatment alone at Mwea (10.72%) and Ngoliba (8.90%) in season 1 (**Figures 4A, B**). Similarly, higher variability was observed in the disease severity among the treatments in season 2 with *H. lixii* F3ST1 (10.71 ± 0.18 and 9.9 ± 1.93%) and *T. asperellum* M2RT4 (19.45 ± 5.22, and 16.75 ± 1.08%) endophytically colonized treatments recording the lowest disease severity rates compared the controls (30.00 **±** 2.28 and 29.22 ± 0.91%) at both Mwea and Ngoliba, respectively (**Figures 4C, D**). The lowest disease severity was recorded in endophytes treated tomato plots compared to buprofezin insecticide treated tomato plots which recorded the highest disease severity (**Figures 4A–D**).

### 3.7 Confirmation of the tomato infectious chlorosis and tomato chlorosis viruses

In additions to the morphological identification through disease symptoms of the pathogens, their identity was also confirmed via molecular tools. For the tomato chlorosis virus, all identity levels percentage similarities ranged from 95.45 – 100% with accession number of LC437668.1 according to BLAST GenBank queries. This high identity rate confirmed test for the virus. However, although the tomato infectious chlorosis virus/disease symptoms were observed in the field, the molecular confirmatory result for the virus based on the protocols described above did not show the virus, which could be due to low titers/status, thus the RNA was not enough for sequencing. It is therefore important that further analysis using stronger and more advanced tools like transcriptomics could also be employed in order to confirm tomato infectious chlorosis virus.

### 3.8 Effect of fungal isolates on the tomato fruits yield

Higher yield was recorded in endophytes treated tomato plants than the controls, and this could be attributed to the vector pest population suppression that resulted to reduced incidence and severity of viral diseases. In season 1, significant differences were observed in the marketable tomato fruits yield among the treatments at Mwea (F_6, 21_ = 7.95; P = 0.0001) and Ngoliba (F_6, 21_ = 6.5; P = 0.0005). On the other hand, there were also significant differences in non-marketable fruits yield among the various treatments at Mwea (F_6, 21_ = 71.06; P < 0.0001) and Ngoliba (F_6, 21_ = 10.53; P < 0.0001) in season 1. The marketable yield was higher in the combination of both endophytes and *M. anisopliae* ICIPE 18 compared to the controls in both sites (**Table 3**). The highest marketable fruits yield was recorded in *H. lixii* F3ST1 + ICIPE 18 treatment in both sites compared to the controls (**Table 3**). Similarly, in season 2, there were also significance differences in the marketable fruits yield (F_6, 21_ = 52.27; P < 0.0001) and non-marketable fruits yield (F_6, 21_ = 480.9; P < 0.0001) among the treatments at Mwea. However, the highest marketable fruits yield was recorded in *H. lixii* F3ST1 + ICIPE 18 treatment compared to the control (**Table 3**). Similar to the trends observed in Mwea, there were also significant variations among the treatments as regard to the marketable fruits yield (F_6, 21_ = 164; P < 0.0001) and non-marketable fruits yield (F_6, 21_ = 198.2; P < 0.0001) at Ngoliba. In addition, the highest marketable tomato fruits yields were recorded in *H. lixii* F3ST1 + ICIPE 18 and *T. asperellum* M2RT4 + ICIPE 18 plots compared to the controls (**Table 3**). There were significant differences in the number of fruits per plant in both at Mwea (F_6, 21_ = 19.2; P < 0.0001) and Ngoliba (F_6, 21_ = 15.2; P < 0.0001) in season 1. Similar trends were also observed in season 2 at Mwea (F_6, 21_ = 39.7.9; P < 0.0001) and Ngoliba (F_6, 21_ = 33.6; P < 0.0001) respectively (**Table 4**).

**Table 3:** Calculated mean yield of *Solanum lycopersicon* fruits in both sites and during the two seasons.

Treatments	Yield (T ha^-1^) (± SE) per season per site							
	Season 1				Season 2			
		Mwea	Ngoliba			Mwea		Ngoliba
	Marketable yield	Non marketable yield	Marketable yield	Non-marketable yield	Marketable yield	Non-marketable yield	Marketable Yield	Non-marketable Yield
*Hypocrea lixii* F3ST1	7.53 ± 0.99abc	2.04 ± 0.82d	10.63 ± 0.46 bc	4.37 ± 0.75bc	8.03 ± 0.37bc	3.04 ± 0.02e	8.53 ± 0.09b	3.37± 0.25d
*Trichoderma asperellum* M2RT4	7.23 ± 0.49abc	3.56 ± 0.51c	9.44 ± 0.73bc	3.81 ± 0.42c	7.98 ± 0.32bc	3.57 ± 0.25d	8.01 ± 0.14b	3.81 ± 0.24d
*Hypocrea lixii* F3ST1+ ICIPE 18	10.23 ± 0.79a	1.29 ± 0.74d	12.18 ± 0.50ab	5.37± 0.76bc	11.23 ± 0.49a	2.29 ± 0.07f	13.08 ± 0.36a	3.04 ± 0.18d
*Trichoderma asperellum* M2RT4+ ICIPE 18	8.66 ± 0.34ab	2.15 ± 0.04d	15.13 ± 0.63a	2.81 ± 0.62c	8.91 ± 0.28b	2.65± 0.23ef	12.41 ± 0.14a	2.91± 0.23d
*Metarhizium. anisoplae* ICIPE 18 alone	6.21 ± 0.50bcd	4.07 ± 0.60c	6.75 ± 0.46 bcd	5.37 ± 0.81bc	6.96 ± 0.16c	4.32 ± 0.05c	7.02 ± 0.30c	4.82 ± 0.26c
Buprofezin insecticide	5.03 ± 0.79cd	6.63 ± 0.36b	5.53 ± 0.01 cd	7.06 ± 0.06b	5.28 ± 0.54d	6.89 ± 0.19b	6.28 ± 0.24c	6.89 ± 0.19b
Control	3.36 ± 0.63d	11.92 ± 0.54a	3.94 ± 0.74 d	10.12 ± 0.78a	2.61 ± 0.34e	12.42 ± 0.15a	2.91 ± 0.45d	12.93 ± 0.37a
df	6,21	6,21	6,21	6,21	6,21	6,21	6,21	6,21
F value	7.96	71.06	6.5	10.53	52.57	480.9	164	198.2
P value	0.0001	< 0.0001	0.0005	< 0.0001	< 0.0001	< 0.0001	< 0.0001	< 0.0001

Means followed by the same letters within a column are not significantly different at P < 0.05.

**Table 4:** Mean number of fruits per plant in both sites and during the two seasons.

Treatments	Number of tomato fruits per plant			
	Season 1		Season 2	
	Mwea	Ngoliba	Mwea	Ngoliba
*Hypocrea lixii* F3ST1	114 ± 0.04ab	124 ± 0.05a	120 ± 0.09cd	129 ± 0.02a
*Trichoderma asperellum* M2RT4	106 ± 0.25bc	111 ± 0.15abc	110 ± 0.05abcd	116 ± 0.05abc
*Hypocrealixii* F3ST1+ ICIPE 18	123 ± 0.30a	130 ± 0.12a	128 ± 0.42a	133 ± 0.20a
*Trichodema asperellum* M2RT4 + ICIPE 18	116 ± 0.56c	108 ± 0.06bcd	118 ± 0.26c	120 ± 0.21b
*Metarhizium anisoplae* ICIPE 18 alone	98 ± 0.40d	79 ± 0.20bc	90 ± 0.22d	93 ± 0.26bc
Buprofezin insecticide	60 ± 0.65cd	52 ± 0.13d	63 ± 0.25e	65 ± 0.11cd
Control	30 ± 0.07e	39 ± 0.07cd	33 ± 0.05ef	40 ± 0.02d
df	6,21	6,21	6,21	6,21
F value	19.2	15.2	39.7	33.6
P value	< 0.0001	< 0.0001	< 0.0001	< 0.0001

Means followed by the same letters within a column are not significantly different at P < 0.05.

## 4 Discussion

The results of this study showed that the two fungal endophytes, *T. asperellum* M2RT4 and *H. lixii* F3ST1 successfully colonized all the tomato plant parts (roots, stems and leaves) at both sites as previously reported by Paradza et al. (2021a) and Agbessenou et al. (2020) in the screenhouse. Similar results were also reported by Mutune et al. (2016) where *T. asperellum* M2RT4, *T. atroviride* F2S21, and *H. lixii* F3ST1 were found to endophytically colonize the various parts of the common bean (*Phaseolus vulgaris*) after artificial seeds inoculation. In addition, Gathage et al. (2016) demonstrated that *H. lixii* F3ST1 successfully colonized all the various parts of *P. vulgaris* under field conditions. Following seed inoculation, *T. harzianum* F2R41, *T. atroviride* F2S21, *T. asperellum* M2RT4 and *H. lixii* F3ST1 were also able to endophytically colonize the various tissues of maize plants (Kiarie et al., 2020; Kinyungu et al., 2023). However, the colonization rates varied significantly as per the plant species and the host plant parts. The ability of these fungal isolates to endophytically colonize these different plant species and with varied colonization rates through artificial inoculation might be due to that the plant species’ physiology and chemistry that may have an impact on the effectiveness of colonization (Akello et al., 2007b; Gathage et al., 2016; Gange et al., 2019). In addition, our results showed that even under field conditions, *T. asperellum* M2RT4 and *H. lixii* F3ST1 were able to outcompete other possible endophytes and systemically colonized *S. lycopersicon* host plants in the different treatments. The ability of these artificially inoculated endophytes to outcompete the other possible endophytes which might already be present in the host plant or in the soil, could also explain their successful colonization in the field (Kambrekar, 2016; Gathage et al., 2016).

Our results showed the highest roots and stems colonization of the tomato plants compared to the leaves at both sites and during the two seasons. These variations could be attributed to endophytic fungus tissue-specificity and their adaptation to specific physiological conditions of the plants (Guo et al., 2008). Similar findings were also reported by Gurulingappa et al. (2010); Posada et al. (2007), and Vega et al. (2008) where the authors observed variations in the extent of colonization of different host plant species and parts. For instance, inoculation of beans with *H. lixii* F3ST1 resulted in greater colonization of the root and stem tissues than leaves, whereas inoculation with *T. atroviride* F2S21 resulted in greater colonization of stem and leaf tissues than roots (Mutune et al., 2016). Similar results were also reported in faba and French beans (Akutse et al., 2013; Paradza et al., 2021a), coffee (Posada et al., 2007), and onion (Muvea et al., 2014). On the other hand, the tomato plants colonized by the tested endophytes have less severe viral diseases symptoms such as leaf area reduction, light downward cupping, pronounced downward or upward leaf chlorosis, leaf mottling, severe mosaic, leaf distortion, crinkling, plant stunting, leaf bunching, and severe necrosis regardless of lower leaves colonization compared to the roots and stems. This could be attributed to endophyte-mediated volatile cues or metabolites which could affect the vector in the transmission of the phytopathogens (Jallow et al., 2008). In addition, feeding on inoculated plants, exposes the insect to secondary metabolites such as terpenoids, isoflavonoids, and isocoumarins that have toxic effects and which inhibit insect performance (Gaggìa et al., 2013; Jaber and Ownley, 2018; Yang et al., 2020). This could be attributed to less severe symptoms expression of the viral diseases despite the level of colonization of the plant parts, since the metabolites production could be stimulated independent of the endophyte colonization levels. The lower colonization in the leaves could also be due to changes in the physiology, hormonal composition, and competition of the plant in the utilization of nutrients with other endophytes as the plant ages (Gaiero et al., 2013; Russo et al., 2019). In addition, it is important to indicate that, endophyte production, scaling, and formulation for widespread usage does not require a major infrastructure and resource investment and this could enable its application and adoption by growers. Furthermore, the scalability and field performance of endophytes could significantly mitigate the environmental effects (due to their endophytic properties) than when using them as entomopathogenic fungi in inundative application; producing reliable outcomes in diverse field settings. On the other hand, endophytes are generally host-specific, meaning one strain may not be universally effective across multiple crops species/varieties.

The effects of endophytes on the greenhouse whitefly number of nymphs in the field were also investigated *in planta* and the results showed significant variations in the number of nymphs among the treatments. However, lower number of nymphs were recorded in endophytically-colonized treatments compared to non-colonized plant plots. This finding supports the results reported by Paradza et al. (2021a) where the authors found that *S. lycopersicum* plants that were endophytically colonized by *H. lixii* F3ST1 and *T. asperellum* M2RT4 successfully suppressed *T. vaporariorum* nymphal development and adult emergence. On the other hand, our results also showed that *M. anisopliae* ICIPE 18 in olive formulation also reduced the number of nymphs, supporting the findings reported by Paradza et al. (2021b) who found that *M. anisopliae* ICIPE 18 in olive oil caused higher *T. vaporariorum* nymphs mortality. The high pathogenicity exhibited by the fungal isolates used in the study has also been reported for other insect orders in several studies. For instance, *M. anisopliae* ICIPE 18 and ICIPE 62 were both reported highly virulent against sweet potato weevil, *Cylas puncticollis* (Bohemen) (Coleoptera: Curculionidae) (Ondiaka et al., 2008), fruitflies, *Ceratitis capitata* (Weidmann) and *C. rosa* var. *fasciventris* (Karsch) (Diptera: Tephritidae) (Dimbi et al., 2013), and legume flower thrips, *Megalurothrips sjostedti* (Trybom) (Thysanoptera: Thripidae) (Ekesi et al., 1998). *Metarhizium anisopliae* ICIPE 18 was also shown to cause mortality of up to 95% to tomato leafminer, *Tuta absoluta* (Meyrick) (Lepidoptera: Gelechiidae) (Agbessenou et al., 2020; Akutse et al., 2020) and stem borer, *Chilo partellus* (Swinhoe) (Lepidoptera: Crambidae) (Maniania, 1992). However, the effect of *M. anisopliae* ICIPE 18 on the vector pest was even more pronounced when combined with the endophytes. The suppression of *T. vaporariorum* population observed in the endophytically colonized plots could be due to the fact that the insect vector pest might be exposed to toxic secondary metabolites such as terpenoids, isoflavonoids, and isocoumarins, which hinder its performance and fitness when feeding on inoculated tomato plants (Gaggìa et al., 2013; Jaber and Ownley, 2018; Yang et al., 2020). In addition, the differences/variations observed in the number of nymphs between the two endophytes *H. lixii* F3ST1 and *T. asperellum* M2RT4 in our study could also be due to endophyte-mediated oviposition preferences via volatile cues (Jallow et al., 2008; Agbessenou et al., 2022). For instance, through host plant-endophyte interactions, some of the endophytes have been reported to induce the systemic release/production of methyl salicylic and jasmonic acid that facilitate plant defense against insect chewers and phloem feeders such as whiteflies (Pappas et al., 2018). In addition, Agbessenou et al. (2022) demonstrated that the endophyte *T. asperellum* M2RT4 activated plant defense mechanisms in tomato plant through both the salicylic and jasmonic acid pathways, which consequently affected the behavior, reproduction traits, and herbivory of *Phthorimaea absoluta* (Meyrick) (Lepidoptera: Gelechiidae). Several studies have also demonstrated the detrimental systemic effects of endophytes in different insect species life-history parameters such as *Liriomyza huidobrensis* (Blanchard) (Diptera: Agromyzidae) (Akutse et al., 2013; Gathage et al., 2016), *Ophiomyia phaseoli* (Tryon) (Diptera: Agromyzidae) (Mutune et al., 2016), *Thrips tabaci* Lindeman (Thysanoptera: Thripidae) (Muvea et al., 2014), *Helicoverpa armigera* (Hübner) (Lepidoptera: Noctuidae) (Jallow et al., 2008; Vidal, 1996), *Tuta absoluta* Lepidoptera: Gelechiidae) (Agbessenou et al., 2020), bean stem maggot *Ophiomyia phaseoli* (Mutune et al., 2016), and greenhouse whitefly *Trialeurodes asperellum* (Paradza et al., 2021a). *Trichoderma* species produce secondary metabolites with biological activity against herbivores (Vinale et al., 2012; Poveda, 2021a) and can influence plant defense chemistry, including the constitutive and induced expression of phytohormones such as jasmonic acid (JA), salicylic acid (SA), abscisic acid (ABA), and ethylene (ET); which are known to regulate plant defense response to herbivory (Jallow et al., 2008; Davis et al., 2013; Kottb et al., 2015; Poveda et al., 2020; Agbessenou et al., 2022). Among these metabolites, JA and SA play an important role in the regulation of cellular immune responses in plants (Singh et al., 2019). Jasmonic acid belongs to a collective group of cyclopentanone plant hormones (jasmonates) that are known to regulate a variety of processes in plant development and inducing insecticidal activities in plants (Black et al., 2003; Degenhardt et al., 2010). In addition, endophytic fungus *T. asperellum* quantitatively and qualitatively alters tomato leaf volatiles composition by enhancing both the SA and JA plant defense pathways. *Trichoderma* also produces an array of hydrolytic enzymes such as glucanases, proteases, lipases and chitinases that degrade the cell walls of pathogenic fungi. The performance of these two endophytes (*H. lixii* F3ST1 and *T. asperellum* M2RT4) in suppressing *T. vaporariorum* in the field showed their efficacy in the control of the pest and their potential integration into whitefly management in tomato cropping systems.

Endophytes also play a role in reducing viral disease incidence and their severity. Our results showed that *H. lixii* F3ST1 and *T. asperellum* M2RT4 significantly reduced the incidence and severity of infectious chlorosis and chlorosis diseases in tomato crops. This could be attributed to the suppression of the vector population, which results to the potential reduction of the virus transmission rates, hence leads to the reduction of incidence and severity of the related diseases. This finding agrees with Birithia et al. (2013) and Kritzman et al. (2001) who reported that endophytically colonized onion plants reduced feeding of thrips and the transmission of Irish Yellow Spot Virus (IYSV) in onions. Similar results were also reported by Lehtonen et al. (2006) who demonstrated that plants (meadow ryegrass, *Lolium pratense*) infected by the endophyte *Neotyphodium uncinatum*, resulted in lower incidence rates of Barley Yellow Dwarf Virus (BYDV) in *L. pretense* compared to endophyte-free plants. This could be due to toxic fungal alkaloids systemically induced by the host plants and which deterred aphids’ vectors transmission of the virus (Lehtonen et al., 2006; Kim et al., 2007; Nyoike and Liburd, 2010; Singh and Kaur, 2020).

Similarly, our results also showed the reduction in the incidence and severity of these viral diseases and consequently reduction of the specific viruses’ transmission in the fungal endophytes inoculated plants compared to the control. This could therefore be attributed to the lower nymphs’ population recorded in the endophytically colonized tomato plants in the two experimental field sites. Our findings are in line with previous studies that reported the use of bacterial and fungal biocontrol agents to provide protection against plant pathogens (Charles et al., 2013; Lacey et al., 2001). For example, it has been reported that the endophyte *H. lixii* F3ST1 inhibited the thrips-transmitted Iris Yellow Spot Virus (IYSP) from replicating in onion plant (Muvea et al., 2018). *Hypocrea lixii* F3ST1 is therefore effective against insect-vectored viral disease (IYSV) through systemic induced resistance via endophyte-thrips-virus mediated interactions and potential production of secondary metabolites; where the IYSV virus infection and transmission were blocked in the endophytically colonized onion plants (Muvea et al., 2018). In addition, Kim et al. (2007) reported that three endophytic isolates of *Lecanicillium* sp. inhibited *Podosphaera fuliginea* (Schlecht.) Pollacci spore production, a plant pathogen that causes powdery mildew. There were several other reports on the ability of endophytes in reducing plant pathogens (Lehtonen et al., 2006; Ownley et al., 2008; Jaber and Salem, 2014; Ownley et al., 2008). Furthermore, the endophyte *T. asperellum* M2RT4 was also reported to trigger the expression of Pathogenesis Related Protein-1 (TomPR1), β-1,3-glucanases (TomPR2), TomloxC, and SIWRKY4 genes, whose main function is to protect host plants against fungal invasion as also observed in our study (Muhorakeye et al., 2024).

Our findings showed *H. lixii* F3ST1 and *T. asperellum* M2RT4 are not only effective in suppressing *T. vaporariorum*, vector of infectious chlorosis and chlorosis viruses, but could also reduce the incidence and severity of the diseases in tomato crops through viral infection and transmission blocking mechanisms. Both endophytes are therefore potential biocontrol agents that could be integrated into sustainable *T. vaporariorum* and chlorosis diseases management in tomato production.

Our results also showed that higher yield was obtained in endophytic treated tomato plants than the control. This could be attributed to the lower *T. vaporariorum* nymphs’ number (pest population suppression) which consequently resulted to reduced viral diseases incidence and severity. In addition, fungal endophytes are known to boost plant growth and activate plant defense mechanisms against various insect pests as observed in this study (Sikora et al., 2008). Our findings concur with those of Kabaluk and Ericsson (2007) and Sánchez-Rodríguez et al. (2018), who observed a significant increase in crop yield of corn and wheat, respectively, from plants colonized by entomopathogenic fungi. These divergent yield results might also be due to the strong endophytic association of specific fungal endophyte strains with host plant species and even plant species varieties (Akutse et al., 2013; Gathage et al., 2016; Jaber and Enkerli, 2017). Similarly, Kabaale et al. (2022) also reported higher marketable yield of tomatoes in plots treated with fungal entomopathogens compared to untreated plots. The authors indicated that the higher marketable fruit yield observed could be due to the suppression of the activity of *P. absoluta*. As a result, there was better photosynthesis, growth, development, flower and fruit retention by tomato plants, and hence better yields, and also less damaged fruits in the treated plots. Our findings also concur with Ndereyimana et al. (2019) who reported improved tomato productivity in plots treated with *M. anisopliae* compared to the control. This also agrees with the findings of Kabaale et al. (2022), that showed a significantly lower fruit yield loss of tomato in *M. anisopliae* ICIPE 20-treated plots compared to untreated plots. On the other hand, Gathage et al. (2016) also reported that incorporation of fungal endophytes *H. lixii* F3ST1 and *B. bassiana* G1LU3 in *P. vulgaris* production system may enhance the management of *Liriomyza* leafminers and greatly improve the crop yield.

The Buprofezin insecticide prevents the production of chitin, which stops the insects from forming a healthy exoskeleton. In addition, the Buprofezin insecticide used in this study did not significantly reduce the *T. vaporariorum* population, viral diseases incidence and severity compared to the tested endophytes. Our results further indicated that it has not significantly improved the tomato yield as compared to the endophytically treated plots. This observation could be attributed to a possible resistance to the insecticide due to the waxy cuticle that limits penetration of the insecticide as reported by Abd-Rabou and Simmons (2012).

In addition, the lowest *T. vaporariorum* nymph population variation observed in our results across the two seasons as well as the TICV and TOCV viral disease incidence and severity rates could also be attributed to some environmental factors such as temperature and rainfall. For instance, whitefly populations tend to decrease during rainy season and vice versa. Thus, the transmission of the viral diseases or pathogens by *T. vaporariorum* greatly depends on their population density. The higher population recorded in our study could be due to higher temperature which can be conducive for population growth. This agrees with Sharma et al. (2017) who reported rapid multiplication of *B. tabaci* during period of higher temperatures while cooler weather, high relative humidity and rainfall can be detrimental to whitefly population and its spread.

In conclusion, the results of this study suggest that the fungal endophytes *H. lixii* F3ST1 and *T. asperellum* M2RT4 suppressed *T. vaporariorum* nymphs’ population and reduced the incidence and severity of infectious chlorosis and chlorosis diseases in tomato plants under field conditions. Both endophytes have therefore high potential to be developed as endophytic-fungal-based biopesticide for the sustainable management of *T. vaporariorum* and the target chlorosis viral diseases. This dual insecticide and fungicide effects of these two fungal endophytes would significantly not only impact tomato growers’ pests and diseases management approaches but also possibly reduce the production cost with higher marketable yield. However, further studies are warranted to confirm or validate these results at large scale field trials and establish the chemical and molecular mechanisms in the endophytically colonized tomato plants by *H. lixii* F3ST1 and *T. asperellum* M2RT4 when elucidating their dual vector and disease control pathways.

## Data Availability

The original contributions presented in the study are included in the article/supplementary material. Further inquiries can be directed to the corresponding author.
